# Late-developing acetabular dysplasia following normal infant hip ultrasound in breech and family-history cohorts: A systematic review

**DOI:** 10.1177/18632521261448943

**Published:** 2026-05-11

**Authors:** Haider Ali Riaz Lakdawala, Yashvi Verma, María Galán-Olleros, Catharine S. Bradley, Kishore Mulpuri, Richard Gardner, Luckshman Bavan

**Affiliations:** 1 Division of Orthopaedic Surgery, The Hospital for Sick Children, Toronto, ON, Canada; 2 Department of Orthopaedic Surgery, BC Children’s Hospital, Vancouver, BC, Canada; 3 Department of Surgery, University of Toronto, Toronto, ON, Canada

**Keywords:** Developmental dysplasia of the hip, late-developing acetabular dysplasia, breech presentation, family history, normal hip ultrasound

## Abstract

**Purpose:**

This systematic review evaluates the reported incidence of late-developing acetabular dysplasia in infants with breech presentation or a positive family history of developmental dysplasia of the hip following normal early ultrasound.

**Methods:**

MEDLINE, Embase, and the Cochrane Library were searched for studies of breech or positive family-history cohorts who underwent early ultrasound and subsequent radiographic follow-up for late-developing acetabular dysplasia. Two reviewers independently screened studies, extracted data, and assessed methodological quality.

**Results:**

Ten studies met inclusion criteria. Across these studies, radiographic follow-up after normal infant ultrasound was incomplete and variably reported, with several cohorts demonstrating substantial attrition. Among infants with a positive family history who underwent radiographic assessment (*n* = 520), no cases of late-developing acetabular dysplasia were reported. In breech cohorts with available follow-up (*n* = 727), reported late-developing acetabular dysplasia rates ranged from 0% to 29%, with higher estimates generally observed in studies with earlier follow-up or more permissive diagnostic thresholds. Management was predominantly observational, and operative treatment was described in one case only.

**Conclusions:**

Persistent acetabular dysplasia after a normal screening ultrasound was not identified in the included family-history cohorts with post-screening imaging. In breech cohorts, reported rates of late-developing acetabular dysplasia vary widely and appear to be influenced by timing of assessment and diagnostic criteria. Methodological heterogeneity and incomplete cohort ascertainment limit interpretation, underscoring the need for prospective studies with standardised definitions and child-level incidence reporting.

**Significance of Study:**

This review highlights critical gaps in the evidence informing management of developemental dysplaisa of the hip following screening and provides a framework for designing definitive prospective studies to guide surveillance practices.

**Level of Evidence:**

Level III.

## Introduction

Developmental dysplasia of the hip (DDH) is one of the most common musculoskeletal conditions in infancy, with incidence estimates ranging from 4 to 23 per 1000 live births.^1^ Timely detection is critical because early treatment leads to substantially better outcomes, reduces the need for surgery, and prevents long-term sequelae such as chronic hip pain, functional limitation, and early-onset osteoarthritis.^2,3^ However, DDH is often clinically silent throughout infancy and early childhood, which has driven the implementation of structured screening programmes worldwide.

Universal ultrasound screening is effective but resource-intensive and may contribute to overdiagnosis and overtreatment.^4,5^ Consequently, many health systems have adopted selective screening strategies that use targeted ultrasound in infants with abnormal neonatal clinical findings or recognised DDH risk factors. Among these risk factors, breech presentation and a positive family history demonstrate the strongest and most consistent associations with DDH and therefore underpin selective screening recommendations in many national guidelines.^6,7^ Despite this, there remains uncertainty regarding the appropriate follow-up of infants with these risk factors who have a normal early hip ultrasound, and whether additional surveillance provides meaningful clinical benefit.

The concern is that these underlying risk factors may continue to influence hip development even after a normal early ultrasound, potentially giving rise to late-developing acetabular dysplasia (LDAD). This has important implications for both follow-up strategies and our understanding of the natural history of DDH. Existing evidence, however, is conflicting. Case reports have described late dislocation in breech infants who previously met criteria for a normal ultrasound examination.^8–10^ Cohort studies attempting to quantify this risk have reported divergent findings: some suggest that infants with normal early ultrasound can be safely discharged without further imaging,^11,12^ while others report higher rates of radiographic dysplasia at later follow-up and advocate ongoing surveillance.^13–15^ Reported incidence varies widely, reflecting differences in follow-up timing and diagnostic thresholds.^16^ This uncertainty has translated into substantial variation in clinical practice, as clinicians attempt to weigh the risk of missed late dysplasia against the potential harms and resource implications of additional imaging and prolonged surveillance. In practice, this ranges from discharge following confirmation of a normal ultrasound to planned radiographic follow-up at variable time points in infancy or early childhood.

This systematic review synthesises available evidence on LDAD in infants with breech presentation or a positive family history who have normal findings on infant hip ultrasound. Our primary objective is to estimate the incidence of LDAD in these risk groups. To aid interpretation of reported incidence estimates, we also describe how studies defined risk factors, confirmed a negative ultrasound screen, the radiographic criteria and timing used to diagnose LDAD, and the subsequent management reported.

## Methods

This systematic review was conducted and reported in accordance with the Preferred Reporting Items for Systematic Reviews and Meta-Analyses (PRISMA) statement.^17^ A prespecified protocol defined the search strategy, eligibility criteria, and approach to data extraction and quality appraisal.

### Data sources and search strategy

A comprehensive search of MEDLINE, Embase, and the Cochrane Library was undertaken on 8 September 2025. The strategy combined keywords and controlled vocabulary (MeSH, Emtree) across three concept domains: DDH, DDH risk factors (specifically breech presentation and positive family history), and infant ultrasound screening. The full search strings for each database are provided in Supplemental Material. Reference lists of included articles were screened to identify additional relevant studies.

### Study selection

**Figure 1.**
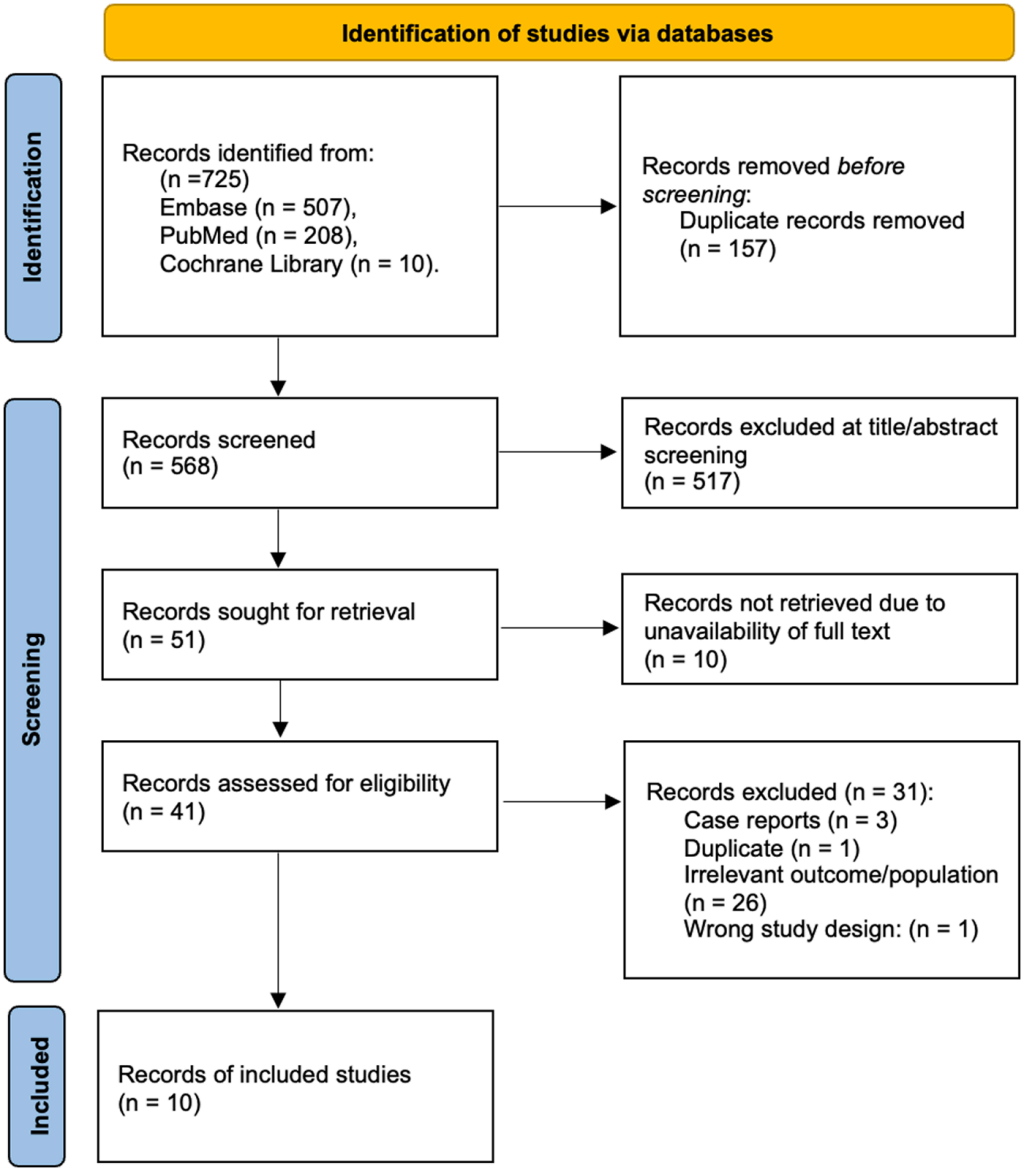
Preferred Reporting Items for Systematic Reviews and Meta-Analyses (PRISMA) 2020 flow diagram for new systematic reviews.^17^ Reproduced under the terms of the Creative Commons Attribution License (CC BY 4.0).

Search results were imported into a citation management database and duplicates removed. Two reviewers independently screened titles and abstracts against the eligibility criteria (Table 1). Full texts of potentially relevant articles were reviewed independently by the same reviewers, with disagreements resolved by discussion or consultation with a third reviewer. Studies reporting mixed-risk cohorts were included only if outcomes for breech and family-history subgroups could be disaggregated or if one risk factor clearly predominated; studies in which subgroup-specific outcomes could not be determined were excluded. Reasons for exclusion at the full-text stage were recorded, and the study selection process is shown in Figure 1.

**Table 1.** Eligibility criteria.

Inclusion
• Cohorts of infants with recognised DDH risk factors (breech presentation and/or positive family history) who underwent hip ultrasound screening in early infancy (<6 months).
• Follow-up pelvic radiographic imaging of hips that were classified as normal at initial ultrasound screening and did not receive treatment.
• Full-text, peer-reviewed articles published in English.
Exclusion
• No recognised diagnostic system for DDH used at infant screening or no criteria provided for defining late-developing acetabular dysplasia.
• Insufficient data to calculate the proportion of children or hips with late-developing acetabular dysplasia in at least one risk-factor subgroup, or mixed-risk cohorts in which outcomes for breech and family-history groups could not be separated and neither risk factor predominated.
• Case reports or case series including fewer than 10 participants.

DDH: Developmental dysplasia of the hip.

### Data extraction and quality appraisal

Data were extracted independently by two reviewers using a standardised data collection tool. Extracted variables included study design, setting and time period, risk-factor subgroup (breech, family history, or both), inclusion criteria, ultrasound classification system and thresholds used at infant screening, age at initial ultrasound, timing at follow-up, diagnostic criteria for LDAD, sample size (children and hips), attrition between screening and follow-up, and reported management of LDAD (observation, bracing, or surgery). When both hip-level (each hip analysed independently) and child-level (each child counted once irrespective of unilateral or bilateral involvement) data were available, child-level estimates were prioritised as the primary analytic unit; studies reporting outcomes only at hip-level were retained and clearly labelled.

Methodological quality and risk of bias were assessed for all included studies using the Joanna Briggs Institute (JBI) Critical Appraisal Checklist for Cohort Studies. Two reviewers independently completed the JBI checklist for each study. Discrepancies were resolved by consensus. Quality appraisal findings were not used to exclude studies but were taken into account when interpreting the heterogeneity and strength of the evidence.

## Results

### Search results and study characteristics

The database search yielded 725 records, of which 10 studies met the eligibility criteria.^18–27^ Six studies primarily evaluated breech cohorts^18,20–24^ and four focused on family-history cohorts.^19,25–27^ All were observational in design: eight retrospective cohort studies and two prospective cohorts, one of which was originally described as a randomised controlled trial but functionally operated as a prospective comparative study.^23^ An overview of included studies and cohort characteristics are provided in Table 2. Methodological quality, assessed using JBI critical appraisal tools, was mixed. Common limitations included inconsistent definitions of risk factors, limited consideration of potential confounders, and variable reporting of follow-up completeness. Strategies to address loss to follow-up were not reported.

**Table 2.** Study cohort characteristics.

Study (first author, year) and risk factor	Risk factor definition	Eligible cohort (*n*)	Attrition rate (%)	Analysed cohort (*n*)	Mean screening age (weeks)	US screening criteria (normal definition)
Breech presentation cohorts						
Antoniak 2021 Retrospective cohort	Born breech	Not defined	Not defined	56	8	α ≥ 60°, FHC ≥ 50%, stability testing
Brusalis 2017 Retrospective cohort	Not defined	Not defined	Not defined	47	6.9 ± 1.7	α ≥ 60°, FHC ≥ 50%
Imrie 2010 Retrospective cohort	Born breech	193	32.1	131	6	Harcke method
Kim 2022 Retrospective cohort	Not defined	742	60.6	292	11.0 ± 6.5	α ≥ 60°, stability testing
Morris 2019 Prospective cohort	Breech at any gestational age	90	24.4	68	4–6^a^	Harcke method
Morris 2021 Prospective cohort	Not defined	476	72.1	133	9.3 ± 3.7	α ≥ 60°, FHC ≥ 50%
Family history cohorts						
Arumilli 2006 Retrospective cohort	First, second or third degree relative	100	11	89	6–8^a^	α ≥ 60°^b^
Aydin and Fatihoglu 2019 Retrospective cohort	Not defined	525	75.0	131	8.3^c^	α ≥ 60°
Tafazal and Flowers 2015 Retrospective cohort	Any relative	159	25.1	119	5.6^c^	α ≥ 60°, FHC ≥ 50%, stability testing
Osarumwense 2007 Retrospective cohort	First or second degree relative	190	4.7	181	6.1	α ≥ 60°, stability testing

FHC: femoral head coverage (%).

a Range.

b 15/100 had α 50–59° (Graf 2a hips).

c Median.

### Cohort size and follow-up completeness

Eight studies reported outcomes at the child level. One study presented both hip- and child-level,^18^ and one reported outcomes only at the hip level, not allowing derivation of child-level proportions.^20^ Several studies explicitly detailed attrition with respect to a defined cohort of eligible infants, while in other cases, it could be inferred from the presented data, with attrition rates as high as 75%.^27^ In contrast, one study defined eligibility on the basis of completed follow-up imaging and did not report the size of the original screened cohort.^20^ Another study reported substantial attrition from an initial screened sample but did not clearly specify what proportion were screened normal and therefore eligible for inclusion within the LDAD analysis.^18^ Consequently, while the included studies collectively described large screened populations, the true number of infants with confirmed normal ultrasound who were eligible for follow-up could not be reliably determined across all studies. A total of 1247 infants had documented radiographic follow-up and therefore contributed to estimates of LDAD; these estimates reflect only those children with follow-up imaging, rather than the entire cohort of infants who screened normal on ultrasound. This analytic cohort comprised 727 infants from predominantly breech cohorts and 520 from family-history cohorts.

### Definitions of risk factors, screening criteria, and LDAD

Among studies providing explicit definitions, two classified breech as presentation at birth,^18,21^ while one defined it as breech at any time in gestation.^23^ Definitions for positive family history ranged from including first- and second-degree relatives^19^ to first-, second-, and third-degree relatives,^25^ or having any relative previously treated for DDH.^26^ Screening typically occurred before 12 weeks of age. Most studies used static Graf methodology, applying an alpha-angle threshold of at least 60° on standardised coronal images, often supplementing this criteria with assessment of femoral head coverage and dynamic sonographic stability. Two studies relied primarily on the Harcke dynamic ultrasound method to exclude DDH and define a normal hip at infant screening.^21,23^

The greatest heterogeneity concerned the definition of LDAD, both in the timing of follow-up imaging and radiographic thresholds. Mean/median follow-up imaging was performed between 5 and 20 months of age. Two studies at the shorter end of this follow-up range reported outcomes using both ultrasound and radiographs; for consistency, only radiographic outcomes were extracted for this review.^26,27^ All studies incorporated the acetabular index (AI) within their radiographic criteria for LDAD, and in most cases, this was the sole metric used. Four studies employed composite definitions that combined AI with additional features, including clinical assessment, side-to-side AI asymmetry, presence or absence of the ossific nucleus, and the relationship of the femoral epiphysis to Hilgenreiner’s and Perkin’s lines.^19,21,25,27^ Additionally, AI thresholds for defining dysplasia varied across studies. Some applied a single absolute cut-off (AI ≥ 30°), while others defined dysplasia as an AI exceeding one or two standard deviations (SD) above age- and sex-specific normative values. Two studies reported incidence estimates using more than one diagnostic threshold.^18,20^

### Incidence and reported clinical outcomes

Given substantial heterogeneity in study design, definitions, follow-up timing, follow-up completeness and outcome reporting, quantitative synthesis was not feasible. Reported incidence estimates, summarised in Table 3, reflect the proportion of infants with LDAD who underwent follow-up radiographic assessment. In family history cohorts, mean/median age at follow-up imaging was generally between 6 and 12 months. Transient radiographic abnormalities were reported in a small number of children at initial follow-up; however, all such cases resolved on subsequent follow-up imaging obtained into the second or third year of life. No child underwent treatment and no cases of persistent acetabular dysplasia were identified on final follow-up of the 520 infants across four family history cohort studies.

**Table 3.** Reported rates of late-developing acetabular dysplasia in high-risk cohorts with normal initial ultrasound.

Study (first author, year) and risk factor	Analysed cohort (*n*)	Mean radiographic follow-up age (months)	Dysplasia definition/criteria	Child level proportion of LDAD at final follow-up (*n*)	Intervention
Breech presentation cohorts					
Antoniak 2021 Retrospective cohort	56	10	AI > 2 SD above mean (Tönnis)^a^	0 (0%)	N/A
Brusalis 2017 Retrospective cohort	47	6.4 ± 0.5	AI > 2 SD above mean (Tönnis)^a^	4 (4.3%) hip level	Brace treatment
Imrie 2009 Retrospective cohort	131	5	Composite radiographic and clinical criteria	38 (29%)	Brace treatment
Kim 2022 Retrospective cohort	292	15.0 ± 1.9	AI > 2 SD above mean (Tönnis)	23 (7.9%)	Observation^b^
Morris 2019 Prospective cohort	68	13 ± 1	AI > 2 SD above mean (Tönnis)	5 (7.4%)	1 acetabuloplasty and capsulorrhaphy 1 arthrogram
Morris 2021 Prospective cohort	133	20.7 ± 6.7	AI > 2 SD above mean (Novais)	3 (2.2%)	Observation
Family history cohorts					
Arumilli 2006 Retrospective cohort	89	6–8^c,d^	Composite radiographic criteria	0 (0%)	Observation^b^
Aydin an Fatihoglu 2019 Retrospective cohort	131	8.2^e^	Composite radiographic criteria	0 (0%)	No LDAD
Tafazal and Flowers 2015 Retrospective cohort	119	6.6^e^	AI > 30°	0 (0%)	Observation^b^
Osarumwense 2007 Retrospective cohort	181	12	AI > 30°	0 (0%)	Observation^b^

LDAD: late-developing acetabular dysplasia; AI: acetabular index; SD: standard deviation.

a Additional AI thresholds were evaluated (>1 SD, Absolute AI > 30°).

b Cases of transient acetabular dysplasia had spontaneous radiographic resolution on further follow-up.

c Range.

d 82/89 infants had follow-up in this range.

e Median.

Reported proportions of LDAD cases in breech cohorts were substantially more variable. Two studies reported low rates comparable to, or below, population-expected thresholds. In one cohort assessed at a mean follow-up of approximately 10 months, no children demonstrated acetabular indices exceeding 2 SD above matched normative population means. Another cohort assessed at a mean follow-up of 20.7 months reported a rate of 2.2% (3/133) using a similar 2 SD threshold. In contrast, the remaining four studies reported higher proportions of radiographic dysplasia. A cohort assessed at a mean follow-up of 6.5 months reported a rate of 4.4%, with affected infants managed with bracing.^20^ The highest reported rate (29%) occurred in a cohort evaluated for LDAD at a mean age of 5 months, using composite radiological and clinical criteria for dysplasia, in which all identified cases were initiated on brace treatment.^21^ A further cohort assessed at a mean follow-up of 13 months reported a rate of 7.4%; two children in this series underwent operative assessment/intervention, including one arthrogram and one procedure described as capsulorrhaphy with acetabuloplasty.^23^

## Discussion

This review synthesises the available evidence on LDAD in infants with breech presentation or a positive family history of DDH who demonstrate normal findings on screening ultrasound. Although LDAD has been reported in several studies, its true frequency within these specific risk groups remains poorly defined. Across the included literature, outcomes were consistently reassuring in family-history cohorts, with no studies reporting persistent radiographic dysplasia or a requirement for treatment following a normal early ultrasound among infants with available follow-up, acknowledging that follow-up completeness and timing varied across studies. In contrast, findings in breech cohorts were more variable, with reported incidence estimates spanning a wide range (0%–29%), reflecting substantial heterogeneity in follow-up timing and diagnostic criteria rather than directly comparable risk estimates, and highlighting ongoing uncertainty regarding the frequency and clinical relevance of LDAD in this population.

Hip ultrasound is a well established and reliable modality for early assessment of DDH, and longitudinal data indicate that normal findings in infancy are strongly predictive of normal acetabular development.^1,5,11,12,28^ Accordingly, infants without concerning clinical features who demonstrate a normal screening ultrasound are typically discharged from care without routine radiographic follow-up.^9^ However, this paradigm has been questioned by reports of LDAD in high-risk cohorts.^20–23^ The extent to which such cases represent true delayed pathology, as opposed to limitations of early assessment, remains uncertain. A recent study not included in this review, owing to non-standardised radiographic assessment, reported no cases of LDAD among high-risk infants and suggested that the quality and thoroughness of the initial ultrasound examination may be critical.^29^ The authors speculated that some cases labelled as late-developing dysplasia in previous cohorts may instead reflect incomplete exclusion of DDH at initial screening. In this context, it is notable that two of the four studies reporting high rates of LDAD relied primarily on the dynamic Harcke method to exclude DDH at screening, which may be less sensitive in detecting the full morphological spectrum of dysplasia compared with Graf-based assessment.^30^

Quantifying the risk of LDAD is further complicated by substantial heterogeneity in the definition of key risk factors. Within DDH screening guidelines, definitions are often restricted to breech at late gestation/delivery, and having a first-degree family member affected by DDH.^7^ In contrast, several included studies applied broader definitions, incorporating breech at any point during gestation or family history extending beyond first-degree relatives. Such inclusivity may dilute apparent risk estimates by incorporating lower-risk infants into cohorts designated as high risk. Attrition presents an additional challenge. Loss to follow-up was common and inconsistently reported, and in some studies, eligibility was defined by completed follow-up imaging rather than by the original screened cohort. This limits confidence in the denominator and introduces uncertainty regarding both the direction and magnitude of potential bias.

The timing and criteria used to define dysplasia introduce further uncertainty. All studies reporting high rates of LDAD assessed infants before walking age, with several evaluating outcomes before 6 months. This represents a period during which physiological variation in acetabular morphology remains common, and across the reviewed literature, hips classified as abnormal at early follow-up frequently normalised on subsequent imaging. Delayed acetabular maturation is well recognised in other DDH contexts, such as following successful Pavlik harness treatment, where many hips meeting dysplasia criteria in late infancy and early childhood normalise spontaneously.^31,32^ The key clinical question, therefore, is whether early radiographic deviations identified in high-risk cohorts persist beyond early childhood, or resolve as part of normal developmental maturation.

This review has several important limitations that warrant consideration. Substantial heterogeneity in study design and outcome reporting precluded quantitative synthesis, necessitating a descriptive approach to summarising reported LDAD risk. This heterogeneity reflects genuine variation in clinical practice and screening pathways, and capturing this variation was central to addressing the review question. In synthesising the available evidence, this review relied on radiographic criteria to categorise both a normal screening ultrasound and subsequent acetabular development. While clinical examination findings and functional features are integral to the diagnosis and management of DDH in routine practice, their definitions, thresholds, and reporting vary widely across the literature and are often incompletely described. As a result, imaging-based measures provided the most consistent and reproducible framework for comparison across studies. We recognise that radiographic findings must always be interpreted in clinical context, but the variability and limited reliability of reported clinical criteria precluded their use as a common benchmark within this review.

In summary, persistent acetabular dysplasia following a normal screening ultrasound was not identified in family-history cohorts undergoing post-screening imaging, although follow-up duration and completeness varied across studies. In breech cohorts, reported rates of LDAD varied widely, reflecting differences in follow-up timing, diagnostic thresholds, and cohort ascertainment. As a result, the current evidence base does not allow reliable estimation of the true child-level incidence or clinical significance of LDAD after a normal ultrasound examination. Prospective studies with standardised eligibility criteria, consistent imaging definitions, and complete follow-up beyond walking age are required to determine whether delayed acetabular development in breech infants represents transient maturation or clinically meaningful dysplasia that warrants surveillance or treatment. Such studies should incorporate age- and sex-adjusted normative radiographic thresholds, careful accounting for attrition, and transparent reporting of management decisions for identified LDAD.

## Supplemental Material

sj-docx-2-cho-10.1177_18632521261448943 – Supplemental material for Late-developing acetabular dysplasia following normal infant hip ultrasound in breech and family-history cohorts: A systematic review Supplemental material, sj-docx-2-cho-10.1177_18632521261448943 for Late-developing acetabular dysplasia following normal infant hip ultrasound in breech and family-history cohorts: A systematic review by Haider Ali Riaz Lakdawala, Yashvi Verma, María Galán-Olleros, Catharine S. Bradley, Kishore Mulpuri, Richard Gardner and Luckshman Bavan in Journal of Children's Orthopaedics

sj-pdf-1-cho-10.1177_18632521261448943 – Supplemental material for Late-developing acetabular dysplasia following normal infant hip ultrasound in breech and family-history cohorts: A systematic review Supplemental material, sj-pdf-1-cho-10.1177_18632521261448943 for Late-developing acetabular dysplasia following normal infant hip ultrasound in breech and family-history cohorts: A systematic review by Haider Ali Riaz Lakdawala, Yashvi Verma, María Galán-Olleros, Catharine S. Bradley, Kishore Mulpuri, Richard Gardner and Luckshman Bavan in Journal of Children's Orthopaedics
