# Supplementary material for: Late-developing acetabular dysplasia following normal infant hip ultrasound in breech and family-history cohorts: A systematic review
Source: J Child Orthop. 2026 May 11;20(4):411–8. doi: 10.1177/18632521261448943 (PMC13161018; doi:10.1177/18632521261448943)
Supplement: sj-docx-2-cho-10.1177_18632521261448943 – Supplemental material for Late-developing acetabular dysplasia following normal infant hip ultrasound in breech and family-history cohorts: A systematic review [file sj-docx-2-cho-10.1177_18632521261448943.docx]

Supplementary File 1. Search strategy applied for all databases for articles written in English.

Pubmed

"Developmental Dysplasia of the Hip"[Mesh] OR "Hip Dislocation, Congenital"[Mesh] OR ((hip[tiab] OR hips[tiab] OR acetabul*[tiab]) AND (dysplas*[tiab] OR dislocat*[tiab] OR subluxat*[tiab] OR instab*[tiab]) AND (congenit*[tiab] OR development*[tiab] OR neonatal[tiab] OR residual[tiab])) OR DDH[tiab] OR "developmental dysplasia of the hip"[tiab] OR "residual acetabular dysplasia"[tiab] OR "residual dysplasia"[tiab] OR "neonatal hip instability"[tiab]

AND

("Breech Presentation"[Mesh] OR breech[tiab] OR "breech birth"[tiab] OR "breech presentation"[tiab] OR "Heredity"[Mesh] OR "Family Characteristics"[Mesh] OR "family history"[tiab] OR "first-degree"[tiab])

AND

("Ultrasonography"[Mesh] OR ultrasound[tiab] OR ultrason*[tiab] OR sonograph*[tiab] OR echogra*[tiab] OR graf[tiab])

Embase

('hip dysplasia'/exp OR 'hip dislocation'/exp OR ((hip OR hips OR acetabul*) NEAR/3 (dysplas* OR dislocat* OR subluxat* OR instab*) NEAR/3 (congenit* OR development* OR neonatal OR residual)):ti,ab,kw OR (DDH OR "developmental dysplasia of the hip" OR "residual acetabular dysplasia" OR "residual dysplasia" OR "neonatal hip instability"):ti,ab,kw)

AND

('breech presentation'/exp OR (breech NEAR/2 (birth OR presentation)):ti,ab,kw OR 'heredity'/exp OR 'family history'/exp OR ("family history" OR first-degree):ti,ab,kw)

AND

('ultrasonography'/exp OR (ultrasound OR ultrason* OR sonograph* OR echogra* OR graf):ti,ab,kw)

Cochrane

[mh "Developmental Dysplasia of the Hip"] OR [mh "Hip Dislocation, Congenital"] OR ("DDH" OR "developmental dysplasia of the hip"):ti,ab,kw OR (hip:ti,ab,kw NEAR/3 (dysplas* OR dislocat* OR subluxat* OR instab*):ti,ab,kw)

AND

[mh "Breech Presentation"] OR (breech:ti,ab,kw NEAR/2 (birth OR presentation):ti,ab,kw) OR [mh "Heredity"] OR ("family history" OR "first-degree" OR "first degree"):ti,ab,kw

AND

[mh "Ultrasonography"] OR (ultrasound OR ultrason* OR sonograph* OR echogra* OR graf):ti,ab,kw
